# Impact of Prophylactic Hydroxychloroquine on People at High Risk of COVID-19: A Systematic Review and Meta-Analysis

**DOI:** 10.3390/jcm10122609

**Published:** 2021-06-13

**Authors:** Adrian V. Hernandez, John Ingemi, Michael Sherman, Vinay Pasupuleti, Joshuan J. Barboza, Alejandro Piscoya, Yuani M. Roman, Charles M. White

**Affiliations:** 1 Health Outcomes, Policy and Evidence Synthesis (HOPES) Group, University of Connecticut School of Pharmacy, Storrs, CT 06269, USA; john.ingemi_iii@uconn.edu (J.I.III); michael.sherman@uconn.edu (M.S.); yuanniroman@gmail.com (Y.M.R.); charles.white@uconn.edu (C.M.W.); 2 Unidad de Revisiones Sistemáticas y Meta-Análisis (URSIGET), Vicerrectorado de Investigación, Universidad San Ignacio de Loyola (USIL), Lima 15024, Peru; jbarbozameca@relaped.com (J.J.B.); alepiscoya@gmail.com (A.P.); 3 Scientific Communications, Cello Health, Yardley, PA 19067, USA; lepiscean@gmail.com; 4 Department of Research Administration, Hartford Hospital, Hartford, CT 06102, USA

**Keywords:** hydroxychloroquine, COVID-19, efficacy, safety, prophylaxis

## Abstract

There are no proven prophylactic interventions for COVID-19. We systematically reviewed the efficacy of prophylactic hydroxychloroquine for COVID-19. Studies evaluating hydroxychloroquine for prophylaxis of COVID-19 were searched in several engines until 8 December 2020. Primary outcomes included RT-PCR positivity, COVID-19 infections (positive RT-PCR or compatible COVID-19 symptoms), and all-cause mortality. Random effects meta-analyses were performed for all outcomes. Five randomized controlled trials (RCTs) (*n* = 5579) and one cohort (*n* = 106) were included. Placebo was the comparator in four RCTs, and usual care in one RCT. Compared to the controls, five RCTs showed that hydroxychloroquine prophylaxis did not reduce RT-PCR positivity (RR 1.01, 95% CI 0.88–1.16), COVID-19 infection (RR 0.98, 95% CI 0.78–1.22), or all-cause mortality (RR 0.73, 95% CI 0.27–1.99). There were no differences of effects by pre- or post-exposure prophylaxis. Prophylaxis with hydroxychloroquine increased the risk of diarrhea, abdominal pain, or vomiting (RR 4.56, 95% CI 1.58–13.19). There were no effects of hydroxychloroquine on other secondary outcomes. Quality of evidence was low to very low for all outcomes. Hydroxychloroquine was not efficacious as a prophylaxis for COVID-19 infections, defined either as RT-PCR positivity or as a composite of RT-PCR positivity or compatible symptoms. Hydroxychloroquine did not reduce all-cause mortality, clinical worsening, or adverse events.

## 1. Introduction

Approximately 11 million people in the United States (US) have been infected with COVID-19 resulting in 500,000 hospitalizations and 252,000 deaths [1,2]. Worldwide, over 55 million COVID-19 cases with over 1.3 million deaths have been reported [1]. For hospitalized patients, the use of remdesivir can lessen the time to recovery [3], while dexamethasone can reduce mortality in the sickest COVID-19 patients [4,5], but there are no proven pharmaceutical treatments to prevent the general public or healthcare workers from contracting the disease. While interim analyses for two vaccines in phase III trials showed ≥ 90% effectiveness in preventing COVID-19 contraction [6,7], the full results are not published. Even with Emergency Use Authorizations, it will be months before all consenting high-risk patients in the US and Europe will receive the two-dose vaccination regimens. For the developing world, it could take markedly longer. In addition, a large swath of the world’s population are reticent to receive COVID-19 vaccinations [8,9]. As such, an effective prophylactic pharmacologic strategy is desperately needed.

As the US and Europe enter this new wave of infections, effective prophylactic therapy may prevent hospitals from being overwhelmed and reduce the morbidity and mortality associated with COVID-19. Some places in the world have recommended the routine use of hydroxychloroquine to prevent COVID-19 [10]. If hydroxychloroquine is efficacious and safe in randomized controlled trials (RCTs), it would be a viable effective prophylactic option because of its low acquisition cost. However, exposing people to the risks of prophylactic hydroxychloroquine without associated benefits and causing drug shortages for patients with autoimmune diseases should not occur unless the benefits are clear. The lack of benefits from hydroxychloroquine in the treatment of hospitalized patients in previous trials [11,12] may not translate into its prophylactic impact. Preventing the onset of COVID-19 with drug therapy might be more successful than treating it later in the disease process.

This systematic review assessed all available controlled studies evaluating the prophylactic use of hydroxychloroquine to prevent COVID-19 infection to identify its benefits and adverse events.

## 2. Materials and Methods

### 2.1. Data Sources and Searches

Three investigators (C.M.W., V.P., and A.V.H.) developed the search strategy, which was revised and approved by the other investigators. We searched the following databases from 1 December 2019 to 8 December 2020: PubMed-MEDLINE, EMBASE-OVID, Scopus, Web of Science, the Cochrane Library, bioRxiv (www.biorxiv.org, accessed on 8 December 2020), Preprints (www.preprints.org, accessed on 8 December 2020), Clinical Trials.gov (accessed in 20 November 2020), the World Health Organization International Clinical Trials Registry Platform (www.who.int/ictrp/en/, accessed on 20 November 2020), and the Chinese Clinical Trials Registry (www.chictr.org.cn, accessed on 20 November 2020). The PubMed search strategy is shown in the Supplemental file.

### 2.2. Study Selection

Controlled studies (RCTs and cohort studies) in any language reporting benefit or harm outcomes from use of hydroxychloroquine on adults at risk for SARS-CoV-2 infection were included. Individuals at risk for SARS-CoV-2 infection included health care workers of hospital-based units (e.g., physicians, nurses, nursing assistants, emergency technicians, and respiratory therapists), household contacts, nursing home workers or residents, or those with a recent history of close-contact exposure to a PCR-confirmed COVID-19 case and absence of COVID-19-like symptoms in the two weeks preceding enrollment. Three investigators (A.V.H., V.P., Y.M.R.) independently screened each record’s title and abstract for potential inclusion. Three investigators (V.P., J.J.B., Y.M.R.) then read the full text of the records whose abstracts had been selected by at least one investigator. Discrepancies were resolved through discussion or by a fourth investigator (A.V.H.).

### 2.3. Outcomes

Primary outcomes were reverse transcription-polymerase chain reaction (RT-PCR)-confirmed for SARS-CoV-2 positivity, the composite COVID-19 infection (RT-PCR positivity or symptoms compatible with new COVID-19 infection), and all-cause mortality. Secondary outcomes included clinical worsening (i.e., hospitalization, intensive care unit (ICU) admission, or need of mechanical ventilation), adverse events, and specific adverse events (e.g., diarrhea, headache, QTc prolongation).

### 2.4. Data Extraction

Two investigators (A.P., J.J.B.) independently extracted the following variables from studies: study setting, country, mean age, proportion of male, type of prophylaxis (pre-exposure vs. post-exposure), hydroxychloroquine dose and duration, type of control and description, additional drug interventions, primary and secondary outcomes, and time of follow up. Discrepancies were resolved through discussion or by a third investigator (A.V.H.).

### 2.5. Risk of Bias Assessment

Two investigators (A.P., J.J.B.) independently assessed risk of bias (RoB) by using the ROBINS-I (Risk of Bias in Non-Randomized Studies of Interventions) tool for cohorts [13] and the Cochrane Risk of Bias 2.0 tool for RCTs [14]; disagreements were resolved by discussion with a third investigator (A.V.H.). RoB per domain and study was described as low, moderate, serious, critical, and no information for cohorts, and as low, some concerns, and high for RCTs.

### 2.6. Statistical Analysis

We reported our systematic review according to the 2009 PRISMA statement [15]. Dichotomous outcomes were described with numbers and proportions, and continuous outcomes with mean and standard deviation or median and interquartile range (IQR). Inverse variance random effect meta-analyses were performed to evaluate the effect of hydroxychloroquine vs. control on outcomes when outcome data were available for at least two RCTs judged to have homogeneous study characteristics. Effects of meta-analyses were reported as relative risks (RR) and their 95% confidence intervals (CIs); a 95% CI including the number 1 in its range meant no difference of outcome effect between hydroxychloroquine and control arms. Data of two arms of hydroxychloroquine from one RCT were combined into one. CIs of effects were adjusted with the Hartung–Knapp method [16], and the between study variance tau^2^ was calculated with the Paule–Mandel method. The treatment arm continuity correction method was used to account for zero outcome events in one or two arms of studies. Heterogeneity of effects among studies was quantified with the I^2^ statistic (an I^2^ > 60% means high heterogeneity). We pre-specified subgroup analyses by type of design (RCTs and cohorts) and by type of prophylaxis (pre-exposure vs. post-exposure); the *p* for an interaction test < 0.05 indicated effect modification by subgroup. The meta package of R 3.5.1 (www.r-project.org, accessed on 17 February 2021) was used for meta-analyses. The quality/certainty of evidence was evaluated using the GRADE methodology, which covers 5 items: risk of bias, inconsistency, indirectness, imprecision, and publication bias [17]. Quality of evidence was evaluated per outcome and described in summary of findings (SoF) tables; GRADEpro GDT was used to create SoF tables [18].

## 3. Results

### 3.1. Selection of Studies

Our comprehensive searches yielded 9378 citations with an additional 927 citations identified through other sources, including backwards citation tracking. After removing duplicates and applying our inclusion and exclusion criteria (Figure S1), we identified five RCTs (*n* = 5579) [19,20,21,22,23] that were eligible for meta-analysis and one cohort study (*n* = 106) [24] which was assessed qualitatively. The cohort by Bhattacharya et al. [23] was published as preprint only.

### 3.2. Characteristics of Included Studies

The general characteristics of the included RCTs and the cohort study are shown in Table 1. Placebo was the comparator in four RCTs [19,21,22,23] with usual care used in one RCT [20] and the cohort study [24]. One RCT evaluated once-a-week vs. twice-a-week hydroxychloroquine regimens vs. placebo [22]. The two pre-exposure RCTs (*n* = 1615) [19,22] used higher total hydroxychloroquine doses (range 10,400 mg to 33,600 mg) and evaluated outcomes after 8 to 12 weeks of prophylaxis. The three post-exposure RCTs (*n* = 3964) [20,21,23] used lower total doses (range 3200 mg to 3800 mg) and evaluated outcomes at 14 days of prophylaxis. The cohort by Bhattacharya et al. evaluated pre-exposure prophylaxis, but dose, duration, and timing of evaluation of outcomes were not reported. Boulware et al. [21] and Rajasingham et al. [22] reported the composite COVID-19 infection as the primary outcome; Mitjà et al. [20] reported the composite of COVID-19 infection as a secondary outcome in a subset of patients with negative RT-PCR positivity at baseline (*n* = 2000, 86% of total evaluated). Clinical worsening was available as hospitalization in Boulware et al. [21] and Barnabas et al. [23] or ICU admission in Rajasingham et al. [22]. Populations were young and mostly healthy; absence of prior comorbidities (i.e., obesity, hypertension, diabetes, and/or coronary disease) ranged between 44 and 83% in RCTs (Table 1).

### 3.3. Risk of Bias of Included Studies

In RCTs, Mitjà et al. [20] and Barnabas et al. [23] had an overall low risk of bias; Abella et al. [19] had overall some concerns of bias (some concerns in the domains of the randomization process and deviation from the intended interventions); and Boulware et al. [21] and Rajasingham et al. [22] had an overall high risk of bias (high risk in the domain measurement of the outcome) (Figures S3 and S4). The Bhattacharya et al. cohort [23] had overall critical risk of bias (critical risk of bias due to confounding).

### 3.4. Prophylactic Effects of Hydroxychloroquine on Primary Outcomes

**Figure 1:**
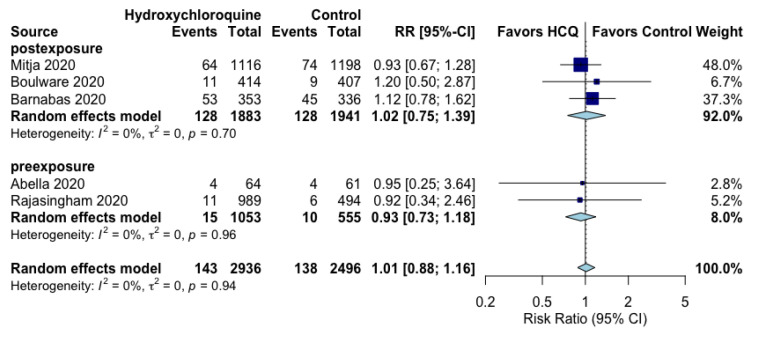
Effect of prophylaxis with hydroxychloroquine on RT-PCR-confirmed SARS-CoV-2 positivity.

**Figure 2:**
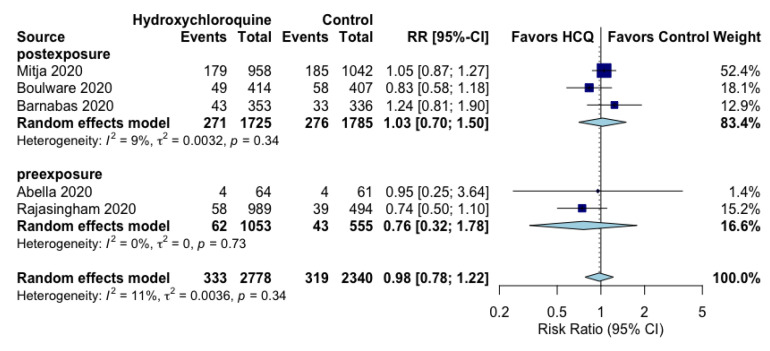
Effect of prophylaxis with hydroxychloroquine on COVID-19 infection (either RT-PCR SARS-Cov-2 positivity or symptoms compatible with COVID-19).

The use of post-exposure or pre-exposure hydroxychloroquine prophylaxis did not reduce the occurrence of RT-PCR-confirmed SARS-CoV-2 positivity (RR 1.01, 95% CI 0.88 to 1.16, I^2^ = 0%) (Figure 1) or the composite of COVID-19 infection (RT-PCR SARS-CoV-2 positivity or symptoms compatible with new COVID-19 infection) (RR 0.98, 95% CI 0.78 to 1.22, I^2^ = 11%) (Figure 2). Subgroup analyses evaluating the prophylactic strategies (post-exposure or pre-exposure) did not substantially alter the direction or magnitude of hydroxychloroquine’s prophylactic efficacy vs. control, and no significant interaction test of *p* values was found.

**Figure 3:**
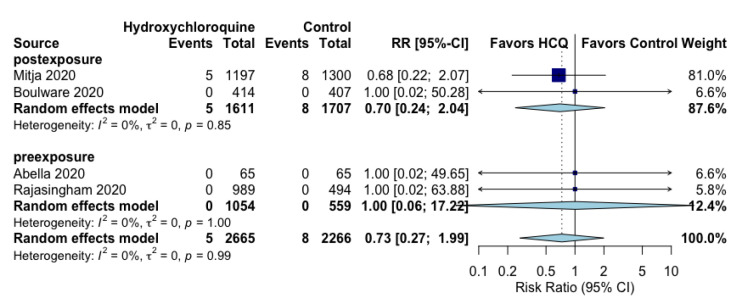
Effect of prophylaxis with hydroxychloroquine on all-cause mortality.

There were very few deaths in the four RCTs. [19,20,21,22] No effect of hydroxychloroquine vs. control was observed on all-cause mortality (RR 0.73; 95% CI 0.27 to 1.99, I^2^ = 0%) (Figure 3). Three RCTs reported zero all-cause mortality events in both arms [19,21,22], and the pooled effect was driven by the events by Mitja et al. [20]. All-cause mortality was a secondary outcome in all studies.

### 3.5. Prophylactic Effects of Hydroxychloroquine on Secondary Outcomes

The use of hydroxychloroquine prophylaxis vs. control did not impact clinical worsening in the three RCTs [21,22,23] evaluating this outcome (RR 1.01, 95% CI 0.17 to 5.92, I^2^ = 0%) (Figure S4). There was no effect of hydroxychloroquine prophylaxis vs. control on severe adverse events in the three RCTs [19,20,23] (RR 0.91, 95% CI 0.48 to 1.75, I^2^ = 0%) (Figure S5). While the direction of effect was towards an increase in the occurrence of adverse events with hydroxychloroquine prophylaxis, no significant effect was seen in the four RCTs [19,20,21,23] (RR 2.79, 95% CI 0.72 to 10.82, I^2^ = 97%) (Figure S6). This effect was driven by the three post-exposure prophylaxis RCTs.

However, there was a significant increase in the occurrence of diarrhea, abdominal pain, or vomiting with hydroxychloroquine prophylaxis vs. control in all five RCTs (RR 4.56, 95% CI 1.58 to 13.19, I^2^ = 95%) (Figure S7). The high statistical heterogeneity in both of these meta-analyses was caused by differences in the magnitude of effects, in particular that of Mitjà et al. [20], but not in the direction of effects among trials. The direction of effect was also toward an increase in headache with hydroxychloroquine prophylaxis vs. control in all five RCTs, but no significant effect was seen (RR 1.38, 95% CI 0.39 to 4.80, I^2^ = 93%) (Figure S8). The resulting high heterogeneity for this outcome was due to differences between studies in both the magnitude and direction of effects.

### 3.6. Cohort Study Description

Bhattacharya et al. [24] conducted a retrospective cohort study of healthcare workers at a tertiary care hospital in India where there was an abrupt cluster outbreak within on-duty personnel. Healthcare workers who voluntarily took prophylactic hydroxychloroquine prior to exposure were compared to those who did not. The investigators did not specify what dose or duration of hydroxychloroquine prophylaxis the active treatment participants received. Most of the evaluated individuals were healthy, as 96% of them did not have prior comorbidities. The primary outcome was the occurrence of laboratory-confirmed COVID-19 infection. Of the 106 participants, 4 out of 54 (7.4%) hydroxychloroquine and 20 out of 52 (38.5%) placebo participants developed laboratory confirmed-COVID-19 (*p* < 0.001). Adverse events were only elucidated in the hydroxychloroquine group, occurred in 29.8% of participants, and were predominantly gastrointestinal.

### 3.7. Quality of Evidence from RCTs

The quality of evidence was low to very low for primary outcomes, including subgroups by prophylaxis type (pre-exposure and post-exposure) (Table 2). The main drivers of poor quality were high risk of bias and imprecision of effects. For secondary outcomes, the quality of evidence was low to very low for all outcomes, and was due to high risk of bias, imprecision, and inconsistency.

## 4. Discussion

Our systematic review found that pre-exposure or post-exposure proplylaxis with hydroxychloroquine did not have an effect on RT-PCR-confirmed SARS-CoV-2 positivity, composite COVID-19 infection (RT-PCR SARS-CoV-2 positivity or symptoms compatible with new COVID-19 infection), or all-cause mortality in RCTs. Hydroxychloroquine prophylaxis was not associated with lower clinical worsening or higher risk of adverse events, except for the composite of diarrhea, abdominal pain, or vomiting. Risk of bias was only low in one of the four RCTs, and the only cohort had a critical risk of bias. Quality of evidence in RCTs was low to very low for all outcomes.

We focused our systematic review on controlled studies where hydroxychloroquine was used specifically for pre- or post-exposure prophylaxis against COVID-19. There are several reasons why we did not allow studies where the experimental group was receiving hydroxychloroquine for the treatment of autoimmune diseases where the subsequent development of COVID-19 was assessed vs. a control group. Patients with autoimmune diseases that are treated with hydroxychloroquine may have a different susceptibility to COVID-19 than those without autoimmune diseases, and the other pharmacotherapeutic options for autoimmune diseases used in the experimental and the control groups might also impact the development of COVID-19 in a positive or negative fashion. Additionally, given hydroxychloroquine’s complex pharmacokinetics (specifically its distribution time into different tissues and its prolonged elimination half-life) [25], the long-term nature of hydroxychloroquine use for autoimmune diseases before exposure may yield tissue concentrations of hydroxychloroquine at the time of exposure that are not achievable with prophylactic use for COVID-19.

In our systematic review, we found a non-significant 2% reduction in the composite outcome COVID-19 infection (RT-PCR-confirmed SARS-CoV-2 positivity or the occurrence of symptoms compatible with COVID-19). While we did not find high heterogeneity of effects across studies, this non-significant reduction was higher in pre-exposure RCTs [19,22] than with post-exposure RCTs [20,21,23] (24% relative risk reduction vs. 3% relative risk increase, respectively). This may be related to the larger total doses of hydroxychloroquine in the pre-exposure prophylaxis RCTs by Abella et al. [19] and Rajasingham et al. [22] and/or the yielding blood and the tissues that are already substantial at the time of exposure, or simply due to chance. In the RCT by Rajasingham et al. [22], 5.9% and 5.9% of those receiving once weekly (HR 0.72, 95% CI 0.44 to 1.16, *p* = 0.18) or twice weekly (HR 0.74, 95% CI 0.46 to 1.19, *p* = 0.22) prophylactic hydroxychloroquine developed composite COVID-19 infection as compared to 7.9% in the placebo group. With double the weekly maintenance dose, the median hydroxychloroquine concentrations in whole blood were as expected; 98 ng/mL (IQR, 82–120) with once-weekly and 200 ng/mL (IQR, 159–258) with twice-weekly hydroxychloroquine dosing. However, hydroxychloroquine concentrations did not differ between those participants who developed a COVID-19-compatible illness or not (154 ng/mL vs. 133 ng/mL, *p* = 0.08). Nasal or pulmonary tissue concentrations of hydroxychloroquine were unfortunately not determined in any trial.

Among the post-exposure RCTs, Bouleware et al. [21] provided a supplementary table where they assessed the impact of hydroxychloroquine on the composite outcome if patients were given prophylactic therapy within one, two, three, or four days of exposure. In their study, participants who enrolled one, two, and three days after exposure had a 48.8%, 28.1%, and 15.9% reduction vs. placebo in new COVID-19 infections, respectively, while those enrolled four days after exposure had a 16.9% increase vs. placebo. Mitjà et al. [20], with only 36.8% of participants receiving prophylaxis ≤ three days after exposure, found a 5% increase in this composite COVID-19 infection with a moderate heterogeneity of effects vs. Boulware et al. [21].

We did not pool the results of the RCTs with the single cohort study that we included in our systematic review, as there are considerably more sources of bias for cohorts [26]. The small pre-exposure cohort study by Bhattacharya et al. [24] found a large and statistically significant reduction in the occurrence of COVID-19. We cannot identify anything unique about Bhattacharya et al. versus the RCTs aside from using a different study method and being conducted in India rather than the US, Canada, or Spain. While the experimental and control groups did not have significant differences in age, gender, or type of exposure, we do not know whether unmeasured variables were similar between groups. Moreover, effects were not adjusted for baseline differences in that limited set of variables, and there was no information to judge the magnitude of immortal bias. In addition, all of the participants were identified via a voluntary online survey, opening up the risk of both sampling and recall bias.

Given the negative effect of hydroxychloroquine prophylaxis that we observed for the RT-PCR-confirmed SARS-CoV-2 positivity and the composite COVID-19 infection, we cannot exclude the possibility that additional RCTs published in the future could generate significant effects on these outcomes. However, those benefits would likely be quite modest and would have to be weighed against the occurrence of adverse events, risk of straining the available hydroxychloroquine drug supply, and the cost and inconvenience of taking this therapy. While serious adverse events were rare, a third to half of all participants did experience adverse events which were predominantly gastrointestinal such as diarrhea, nausea, cramping, loose stools, and vomiting. We individually meta-analyzed for serious adverse events; any adverse events; the composite of diarrhea, abdominal pain, or vomiting; and headache, with the composite of diarrhea, abdominal pain, or vomiting showing a statistically significant increase, and the other endpoints only showing non-statistically significant effects with hydroxychloroquine prophylaxis.

Diverting the hydroxychloroquine drug supply for the prevention or treatment of COVID-19 has already negatively impacted patients with autoimmune diseases. Of the 3872 patients taking hydroxychloroquine or chloroquine for autoimmune diseases in one study [27], 21%, 27%, 7%, and 2% of patients in South-East Asia, Africa, North and South America, and Europe, respectively, reported running out of medication due to drug shortages in the COVID-19 era. While hydroxychloroquine is inexpensive per dose, providing it to millions of healthcare workers across the globe has a substantial cost associated with it. Finally, it is known that people with an appreciable pill burden are frequently less adherent to chronic medications and more prone to adverse outcomes as a result [28]. Given all of these factors, the balance of benefits to harms is unfavorable for the prophylactic use of hydroxychloroquine. Hydroxychloroquine should clearly not be touted as a viable alternative to vaccines to prevent COVID-19.

Our study had several limitations. First, all-cause mortality had a very low incidence across studies, and was usually a secondary outcome. We used the treatment arm continuity correction method to account for zero all-cause mortality events; we also assessed effects of hydroxychloroquine on all-cause mortality with Mantel–Haenzel fixed effects models and found no differences with our main analyses. Second, all outcomes had low or very low quality of evidence, mainly driven by high or some concerns of bias, and imprecision of effects. Third, we assessed a few individual adverse events due to scarcity of reporting across RCTs; only gastrointestinal adverse events (i.e., diarrhea, abdominal pain, or vomiting) and headache could be analyzed. Finally, there were several sources of heterogeneity across studies. Pre-exposure RCTs were conducted on health care workers (HCWs), and post-exposure RCTs were conducted on both HCWs and close contacts. To account for different types of patients and prophylaxis, and different follow-up times across studies, we primarily planned and performed stratified meta-analyses by type of prophylaxis (pre-exposure studies had 8–12 weeks follow up, and post-exposure studies had 2 weeks follow up) for all primary and secondary outcomes. Importantly, we did not combine the cohort with the RCTs, as these study designs are very different, and patients overall were young and with a few comorbidities across all studies.

## 5. Conclusions

There was no effect of prophylaxis with hydroxychloroquine vs. placebo or usual care on RT-PCR-confirmed SARS-CoV-2 positivity, on the composite COVID-19 infection (RT-PCR SARS-CoV-2 positivity or having symptoms consistent with COVID-19), or on all-cause mortality. Hydroxychloroquine prophylaxis did not improve clinical worsening (i.e., hospitalizations or ICU admission) or increased serious adverse events or adverse events, except for the composite of diarrhea, abdominal pain, or vomiting. The quality of evidence was low to very low for all outcomes. The balance of expected benefits to harms for prophylactic hydroxychloroquine is currently unfavorable and cannot be recommended at this time.

## Figures and Tables

**Table 1:** Baseline characteristics of included randomized controlled trials.

Author, Year [ref]/Type of Study/Registration	Objective	Sample Randomized (Arm Sizes), Country(ies), Population	Overall Key Patient Characteristics	Intervention	Comparison	Outcomes	Follow-Up Time
Abella, 2020 [19]/Parallel RCT/NCT04329923	To evaluate the effect of daily HCQ to prevent SARS-CoV-2 infection in hospital-based HCWs over 8 weeks of exposure via RT-PCR testing of NP swabs and serologic antibody testing.	132 (HCQ = 66, placebo = 66), USA, HCWs who worked > 20 h/week in hospital-based units, had no known history SARS-CoV-2 infection, did not have symptoms suggestive of COVID-19 in the 2 weeks before enrollment, including cough, fever, or shortness of breath Physicians, nurses, certified nursing assistants, emergency technicians, and respiratory therapists were eligible	Median age (range): 33 (20–66) y Male: 31% No prior disease: 71%	200 mg of HCQ, 3 times a day with food, for 8 weeks. (33,600 mg total)	Custom-molded identically sized and shaped microcrystalline cellulose placebo tablets for 8 weeks	Primary: SAR-CoV-2 positive status via NP swab. Secondary: adverse events, serological antibody positivity for either nucleocapsid or spike protein antigens, ECG changes after 4 weeks of treatment, and clinical outcomes for any participants who became SARS-CoV-2 positive and/or developed COVID-19 symptoms.	8 weeks
Mitjà, 2020 [20]/Cluster RCT/NCT04304053	To investigate the efficacy/safety of HCQ to prevent secondary PCR-confirmed symptomatic COVID-19 and SARS-CoV-2 infection in contacts exposed to a PCR-positive COVID-19 case.	2314 (HCQ = 1116, Usual care = 1198), Spain, Age ≥ 18 years, either a healthcare worker, a household contact, a nursing home worker, or a nursing home resident Recent history of close contact exposure to a PCR-confirmed COVID-19 case (i.e., > 15 min within two meters, up to seven days before enrolment) Absence of COVID-19-like symptoms in the two weeks preceding enrollmentInclusion was irrespective of baseline PCR result.	Mean age (SD): 49 (19) yMale: 27% No prior disease: 44%	800 mg HCQ on day 1 followed by 400 mg once daily for 6 days (3200 mg total)	Usual care (unspecified)	Primary: Confirmed COVID-19 episode, defined as symptomatic illness (> = 1 among: fever, cough, difficulty breathing, myalgia, headache, sore throat, new olfactory and taste disorder(s), or diarrhea) and a positive SARS-CoV-2 RT-PCR test. Secondary: SARS-CoV-2 infection (either RT-PCR detection of SARS-CoV-2 in a NP specimen or the presence of any of the aforementioned symptoms compatible with COVID-19).	14 days (infection)/ 28 days (adverse events)
Boulware, 2020 [21]/Parallel RCT/NCT04308668	To determine the efficacy of HCQ as post-exposure prophylaxis, to prevent symptomatic infection after exposure to COVID-19.	821 (HCQ = 414), Placebo = 407), USA and Canada. All participants were asymptomatic at enrollment, who had household or occupational exposure to a person with confirmed COVID-19	Median age (IQR): 40 (33–50) y Male: 48% No prior disease: 73%	HCQ 800 mg (4 tablets) once, then 600 mg 6 to 8 h later, then 600 mg daily for 4 more days (3800 mg total).	Matching placebo folate tablets, identical regimen as HCQ.	Primary: Symptomatic illness confirmed by a positive molecular assay or COVID-19 related symptoms within 14 days. Secondary: hospitalization for COVID-19 or death, PCR-confirmed SARS-CoV-2 infection, COVID-19 symptoms, discontinuation of intervention owing to any cause, and severity of symptoms (if any) at days 5 and 14.	14 days
Rajasingham, 2020 [22]/Parallel RCT / No registration	To explore the potential of HCQ as a pre-exposure prophylaxis for COVID-19 in HCW in a tertiary care hospital.	1483 (HCQ OW = 494, HCQ TW = 495, Placebo = 494), USA and Canada, HCW > = 18 years with ongoing exposure to persons with COVID-19, working at ER, ICU, hospital wards, and first responders.	Median age (IQR): 41 (34–49) y Male: 49% No prior disease: 66%	HCQ loading dose of 400 mg (two tablets) twice separated by 6–8 h followed by (I) 400 mg OW for 12 w (5600 mg total) or (II) 400 mg TW for 12 w (10,400 mg total).	Matching Placebo: loading dose of two tablets followed by two tablets OW or TW for 12 w. Placebos are combined in analyses (randomization was 2:2:1:1).	Primary: COVID-19 infection confirmed by PCR or probable compatible illness. Secondary: confirmed SARS-CoV-2 infection, possible COVID-19, and hospitalization, death, or other adverse events.	12 weeks
Barnabas, 2020 [23]/Cluster RCT/NCT04328961	To test HCQ as post-exposure prophylaxis for SARS-CoV-2 infection	829 (HCQ = 407, Placebo = 422), USA, Individuals able to provide informed consent, were 18 to 80 years of age, had close contact with a person (index) with recent known SARS-CoV-2 infection, had exposure within the prior 96 h, were able to conduct study visits via telehealth, and were not planning to take hydroxychloroquine outside the study.	Median age (IQR): 39 (27–51) y Male: 40% No prior disease (Metabolic disease): 83%	HCQ 400 mg/d orally for 3 days, then 200 mg/d orally for an additional 11 days (3400 mg total)	Ascorbic acid (500 mg/d orally for 3 days, then 250 mg/d orally for 11 days) as a placebo equivalent.	Primary: PCR-confirmed incident SARS-CoV-2 infection through day 14 among persons who were SARS-CoV-2 negative at enrollment. Secondary: PCR-confirmed incident SARS-CoV-2 infection at 28 days, symptomatic COVID-19 disease per CDC definition at 14 days.	14 days
Bhattacharya, 2020 [24]/Cohort/ No registration	To explore the potential of HCQ as a pre-exposure prophylaxis for COVID-19 in health care workers in a tertiary care hospital.	106 (HCQ = 54, Non-HCQ = 52), India, HCW who worked at Medical College in India dealing with COVID-19 patients in the first two weeks of May 2020. In the given period, a cluster outbreak of cases amongst HCWs in this hospital had occurred—with about 28 HCW testing positive over a period of two weeks.	Mean age (SD): 27.1 (5.8) y Male: 49% No prior disease (comorbidities): 96%	HCQ only. No doses or duration reported. HCW were voluntarily on Pre-exposure HCQ prophylaxis	Non-HCQ; No other details.	Primary: RT-PCR positive COVID-19 infection. Secondary: adverse events.	Not specified

Ref: reference; HCQ: Hydroxychloroquine; Non-HCQ: No hydroxychloroquine; HCW: Health care worker; RT-PCR: Reverse transcription-polymerase chain reaction; NP: Nasopharyngeal; ECG: electrocardiogram; hx: History; OW: once weekly; TW: twice weekly; ER: emergency room; ICU: Intensive Care Unit; w: week; IQR: Interquartile range.

**Table 2:** Summary of findings table for the effects of prophylactic hydroxychloroquine vs. control in individuals at high risk of COVID-19.

Outcomes	Anticipated Absolute Effects * (95% CI)		Relative Effect (95% CI)	№ of Participants (Studies)	Certainty of the Evidence(GRADE)
	Risk with Control	Risk with Hydroxychloroquine			
SARS-CoV-2 Positivity assessed with: RT-PCR follow up: range 2 weeks to 12 weeks	6 per 100	6 per 100 (5 to 6)	RR 1.01 (0.88 to 1.16)	5432 (5 RCTs)	⨁⨁◯◯ LOW ^a^
SARS-CoV-2 positivity (pre-exposure) assessed with: RT-PCR follow up: range 8 weeks to 12 weeks	2 per 100	2 per 100 (1 to 2)	RR 0.93 (0.73 to 1.18)	1608 (2 RCTs)	⨁⨁◯◯ LOW ^b^
SARS-CoV-2 positivity (post-exposure) assessed with: RT-PCR follow up: mean 14 days	7 per 100	7 per 100 (5 to 9)	RR 1.02 (0.75 to 1.39)	3824 (3 RCTs)	⨁⨁◯◯ LOW ^c^
Composite COVID-19 Infection assessed with: RT-PCR positivity or symptoms compatible with COVID-19 follow up: range 2 weeks to 12 weeks	14 per 100	13 per 100 (11 to 17)	RR 0.98 (0.78 to 1.22)	5118 (5 RCTs)	⨁⨁◯◯ LOW ^a^
Composite COVID-19 infection (pre-exposure) assessed with: RT-PCR positivity or symptoms compatible with COVID-19 follow up: range 8 weeks to 12 weeks	8 per 100	6 per 100 (2 to 14)	RR 0.76 (0.32 to 1.78)	1608 (2 RCTs)	⨁◯◯◯ VERY LOW ^b^^,^^d^
Composite COVID-19 infection (post-exposure) assessed with: RT-PCR positivity or symptoms compatible with COVID-19 follow up: mean 14 days	15 per 100	16 per 100 (11 to 23)	RR 1.03 (0.70 to 1.50)	3510 (3 RCTs)	⨁⨁◯◯ LOW ^c^^,^^e^
All-cause mortalityfollow up: range 2 weeks to 12 weeks	0 per 100	0 per 100 (0 to 1)	RR 0.73 (0.27 to 1.99)	4931 (4 RCTs)	⨁◯◯◯ VERY LOW ^f^^,^^g^
Clinical worseningassessed with: hospitalization or ICU admissionfollow up: range 2 weeks to 12 weeks	0 per 100	0 per 100 (0 to 1)	RR 1.01 (0.17 to 5.92)	3133 (3 RCTs)	⨁◯◯◯ VERY LOW ^f^^,^^h^
Severe adverse vents follow up: range 2 weeks to 8 weeks	1 per 100	1 per 100 (1 to 2)	RR 0.91 (0.48 to 1.75)	3456 (3 RCTs)	⨁⨁◯◯ LOW ^i^^,^^j^
Adverse events assessed with: Any type of adverse event follow up: range 2 weeks to 8 weeks	9 per 100	26 per 100 (7 to 100)	RR 2.79 (0.72 to 10.82)	4156 (4 RCTs)	⨁◯◯◯ VERY LOW ^k^^,^^l^^,^^m^
Diarrhea, abdominal pain, or vomiting follow up: range 2 weeks to 12 weeks	4 per 100	17 per 100 (6 to 49)	RR 4.56 (1.58 to 13.19)	5639 (5 RCTs)	⨁◯◯◯ VERY LOW ^a^^,^^n^^,^^o^
Headache follow up: range 2 weeks to 12 weeks	2 per 100	3 per 100 (1 to 11)	RR 1.38 (0.39 to 4.80)	5639 (5 RCTs)	⨁◯◯◯ VERY LOW ^a^^,^^p^^,^^q^

a. Risk of bias: high risk of bias in Boulware et al. and Rajasingham et al.; both in the domain of measurement of outcome; some concerns of bias in Abella et al. in domains of randomization process and deviation from the intended interventions. b. Risk of bias: high risk of bias in Rajasingham et al. in the domain measurement of the outcome and some concerns of bias in Abella et al. in domains of randomization process and deviation from the intended interventions. c. Risk of bias: high risk of bias in Boulware et al. in the domain of measurement of the outcome. d. Imprecision: 95% CI of the RR was 0.32 to 1.78 e. Imprecision: 95% CI of the RR was 0.70 to 1.50. f. Risk of bias: high risk of bias in Boulware et al. and Rajasingham et al. both in the domain of measurement of outcome. g. Imprecision: 95% CI of the RR was 0.27 to 1.99. h. Imprecision: 95% CI of the RR was 0.17 to 5.92. i. Risk of bias: Some concerns of bias in Abella et al. in domains of randomization process and deviation from intended interventions. j. Imprecision: 95% CI of RR was 0.48 to 1.75. k. Risk of bias: high risk of bias in Boulware et al. in the domain of measurement of the outcome, and some concerns of bias in Abella et al. in domains of randomization process and deviation from intended interventions. l. Inconsistency: I2 = 97%. m. Imprecision: 95% CI of the RR was 0.72 to 10.82. n. Inconsistency: I2 = 95%. o. Imprecision: 95% CI of the RR was 1.58 to 13.19. p. Inconsistency: I2 = 93%. q. Imprecision: 95% CI of the RR was 0.39 to 4.80.

## Supplementary Materials

The following are available online at https://www.mdpi.com/article/10.3390/jcm10122609/s1 file, Supplemental Methods: PubMed search strategy; Figure S1: Flowchart of study selection; Figure S2: Risk of bias assessment of five randomized controlled trials; Figure S3: Detailed risk of bias assessment per randomized controlled trial; Figure S4: Effect of prophylaxis with hydroxychloroquine on clinical worsening; Figure S5: Effect of prophylaxis with hydroxychloroquine on severe adverse events; Figure S6: Effect of prophylaxis with hydroxychloroquine on adverse events; Figure S7: Effect of prophylaxis with hydroxychloroquine on diarrhea, abdominal pain, or vomiting; Figure S8: Effect of prophylaxis with hydroxychloroquine on headache.

## References

[1] Worldometer Worldometer’s COVID-19 Dataset. https://www.worldometers.info/coronavirus/.

[2] Johns Hopkins University The COVID Tracking Project. https://covidtracking.com/data/national/hospitalization.

[3] Beigel J.H., Tomashek K.M., Dodd L.E., Mehta A.K., Zingman B.S., Kalil A.C., Hohmann E., Chu H.Y., Luetkemeyer A., Kline S. (2020). Remdesivir for the Treatment of Covid-19-Final Report. N. Engl. J. Med..

[4] Horby P., Lim W.S, Emberson J.R., Mafham M., Bell J.L., Linsell L., Staplin N., Brightling C., Ustianowski A., RECOVERY Collaborative Group (2020). Dexamethasone in Hospitalized Patients with Covid-19. N. Engl. J. Med..

[5] Tomazini B.M., Maia I.S., Cavalcanti A.B., Berwanger O., Rosa R.G., Veiga V.C., Avezum A., Lopes R.D., Bueno F.R., Silva M.V.A. (2020). Effect of Dexamethasone on Days Alive and Ventilator-Free in Patients With Moderate or Severe Acute Respiratory Distress Syndrome and COVID-19: The CoDEX Randomized Clinical Trial. JAMA.

[6] Pfizer and BionTech Press Release Pfizer and BionTech Announce Vaccine Candidate Against COVID-19 Achieved Success in First Interim Analysis from Phase III Study. 11/9/2020. https://www.pfizer.com/news/press-release/press-release-detail/pfizer-and-biontech-announce-vaccine-candidate-against.

[7] National Institutes of Health Promising Interim Results from Clinical Trial of NIH-Moderna COVID-19 Vaccine. 11/16/2020. https://www.nih.gov/news-events/news-releases/promising-interim-results-clinical-trial-nih-moderna-covid-19-vaccine.

[8] Tyson A., Johnson C., Funk C.U.S. Public Now Divided Over Whether To Get COVID-19 Vaccine. Concerns about the Safety and Effectiveness of Possible Vaccine, Pace of Approval Process. Pew Research Group. 9/17/2020. https://www.pewresearch.org/science/2020/09/17/u-s-public-now-divided-over-whether-to-get-covid-19-vaccine/.

[9] Lazarus J.V., Ratzan S.C., Palayew A., Gostin L.O., Larson H.J., Rabin K., Kimball S., El-Mohandes A. (2021). A global survey of potential acceptance of a COVID-19 vaccine. Nat. Med..

[10] COVID Analysis Early Treatment with Hydroxychloroquine: A Country-Based Analysis. 11/4/2020. https://hcqtrial.com/.

[11] Hernandez A.V., Roman Y.M., Pasupuleti V., Barboza J.J., White C.M. (2020). Hydroxychloroquine or Chloroquine for Treatment or Prophylaxis of COVID-19: A Living Systematic Review. Ann. Intern. Med..

[12] Hernandez A.V., Roman Y.M., Pasupuleti V., Barboza J.J., White C.M. (2020). Update Alert 3: Hydroxychloroquine or Chloroquine for the Treatment or Prophylaxis of COVID-19. Ann. Intern. Med..

[13] Sterne J.A., Hernán M.A., Reeves B.C., Savović J., Berkman N.D., Viswanathan M., Henry D., Altman D.G., Ansari M.T., Boutron I. (2016). ROBINS-I: A tool for assessing risk of bias in non-randomised studies of interventions. BMJ.

[14] Sterne J.A.C., Savović J., Page M.J., Elbers R.G., Blencowe N.S., Boutron I., Cates C.J., Cheng H.Y., Corbett M.S., Eldridge S.M. (2019). RoB 2: A revised tool for assessing risk of bias in randomised trials. BMJ.

[15] Moher D., Liberati A., Tetzlaff J., Altman D.G., PRISMA Group (2009). Preferred reporting items for systematic reviews and meta-analyses: The PRISMA statement. PLoS Med..

[16] Hartung J., Knapp G. (2001). A refined method for the meta-analysis of controlled clinical trials with binary outcome. Stat. Med..

[17] Balshem H., Helfand M., Schünemann H.J., Oxman A.D., Kunz R., Brozek J., Vist G.E., Falck-Ytter Y., Meerpohl J., Norris S. (2011). GRADE guidelines: 3. Rating the quality of evidence. J. Clin. Epidemiol..

[18] GRADEpro GDT: GRADEpro Guideline Development Tool [Software] McMaster University, 2020 (Developed by Evidence Prime, Inc.). gradepro.org.

[19] Abella B.S., Jolkovsky E.L., Biney B.T., Uspal J.E., Hyman M.C., Frank I., Hensley S.E., Gill S., Vogl D.T., Maillard I. (2021). Efficacy and Safety of Hydroxychloroquine vs Placebo for Pre-exposure SARS-CoV-2 Prophylaxis Among Health Care Workers: A Randomized Clinical Trial. JAMA Intern. Med..

[20] Mitjà O., Corbacho-Monné M., Ubals M. A, Alemany A., Suñer C., Tebé C., Tobias A., Peñafiel J., Ballana E., Pérez C.A. (2021). Cluster-Randomized Trial of Hydroxychloroquine for Prevention of Covid-19. N. Engl. J. Med..

[21] Boulware D.R., Pullen M.F., Bangdiwala A.S., Pastick K.A., Lofgren S.M., Okafor E.C., Skipper C.P., Nascene A.A., Nicol M.R., Abassi M. (2020). Randomized Trial of Hydroxychloroquine as Postexposure Prophylaxis for Covid-19. N. Engl. J. Med..

[22] Rajasingham R., Bangdiwala A.S., Nicol M.R, Skipper C.P., Pastick K.A., Axelrod M.L., Pullen M.F., Nascene A.A., Williams D.A., Engen N.W. (2021). Hydroxychloroquine as pre-exposure prophylaxis for Coronavirus Diseases 2019 (COVID-19) in healthcare workers: A randomized trial. Clin. Infect. Dis..

[23] Barnabas R.V., Brown E.R., Bershteyn A., Stankiewicz Karita H.C., Johnston C., Thorpe L.E., Kottkamp A., Neuzil K.M., Laufer M.K., Deming M. (2021). Hydroxychloroquine as Postexposure Prophylaxis to Prevent Severe Acute Respiratory Syndrome Coronavirus 2 Infection: A Randomized Trial. Ann. Intern. Med..

[24] Bhattacharya R., Chowdhury S., Mukherjee R. Pre Exposure Hydroxychloroquine Prophylaxis for Covid-19 in Healthcare Workers: A Retrospective Cohort. medRxiv2020.06.09.20116806.

[25] Morrisette T., Lodise T.P., Scheetz M.H., Goswami S., Pogue J.M., Rybak M.J. (2020). The Pharmacokinetic and Pharmacodynamic Properties of Hydroxychloroquine and Dose Selection for COVID-19: Putting the Cart Before the Horse. Infect. Dis. Ther..

[26] McKenzie J.E., Brennan S.E., Ryan R.E., Thomson H.J., Johnston R.V., Thomas J., Higgins J.P.T., Thomas J., Chandler J., Cumpston M., Li T., Page M.J., Welch V.A. (2020). Chapter 3: Defining the criteria for including studies and how they will be grouped for the synthesis. Cochrane Handbook for Systematic Reviews of Interventions.

[27] Sirotich E., Kennedy K., Surangiwala S. Antimalarial Drug Shortages During the COVID-19 Pandemic: Results from the Global Rheumatology Alliance Patient Experience Survey. American College of Neurology Abstracts 2020. https://acrabstracts.org/abstract/antimalarial-drug-shortages-during-the-covid-19-pandemic-results-from-the-global-rheumatology-alliance-patient-experience-survey/.

[28] Sutton S., Magagnoli J., Hardin J.W. (2016). Impact of Pill Burden on Adherence, Risk of Hospitalization, and Viral Suppression in Patients with HIV Infection and AIDS Receiving Antiretroviral Therapy. Pharmacotherapy..

