# Supplementary material for: Impact of Prophylactic Hydroxychloroquine on People at High Risk of COVID-19: A Systematic Review and Meta-Analysis
Source: J Clin Med. 2021 Jun 13;10(12):2609. doi: 10.3390/jcm10122609 (PMC8231762; doi:10.3390/jcm10122609)
Supplement: Supplementary file 1 [file jcm-10-02609-s001.zip › jcm-1235010-supplementary.pdf]

**Supplemental file**

**Supplemental Methods: PubMed Search Strategy**

**Figure S1:** Flowchart of study selection

**Figure S2:** Risk of bias assessment of five randomized controlled trials

**Figure S3:** Detailed risk of bias assessment per randomized controlled trial

**Figure S4:** Effect of prophylaxis with hydroxychloroquine on clinical worsening

**Figure S5:** Effect of prophylaxis with hydroxychloroquine on severe adverse events

**Figure S6:** Effect of prophylaxis with hydroxychloroquine on adverse events

**Figure S7:** Effect of prophylaxis with hydroxychloroquine on diarrhea, abdominal pain or vomiting.

**Figure S8:** Effect of prophylaxis with hydroxychloroquine on headache

**Supplemental Methods: PubMed Search strategy**

("hydroxychloroquine"[MeSH Terms] OR "hydroxychloroquine"[All Fields] OR ("chloroquin"[All Fields] OR "chloroquine"[MeSH Terms] OR "chloroquine"[All Fields] OR "chloroquine s"[All Fields] OR "chloroquines"[All Fields])) AND ("severe acute respiratory syndrome coronavirus 2"[Supplementary Concept] OR "severe acute respiratory syndrome coronavirus 2"[All Fields] OR "ncov"[All Fields] OR "2019 ncov"[All Fields] OR "covid 19"[All Fields] OR "sars cov 2"[All Fields] OR (("coronavirus"[All Fields] OR "cov"[All Fields]) AND 2019/11/01:3000/12/31[Date - Publication]) OR ("severe acute respiratory syndrome coronavirus 2"[Supplementary Concept] OR "severe acute respiratory syndrome coronavirus 2"[All Fields] OR "sars cov 2"[All Fields]))

**Figure S1.** Flowchart of study selection.

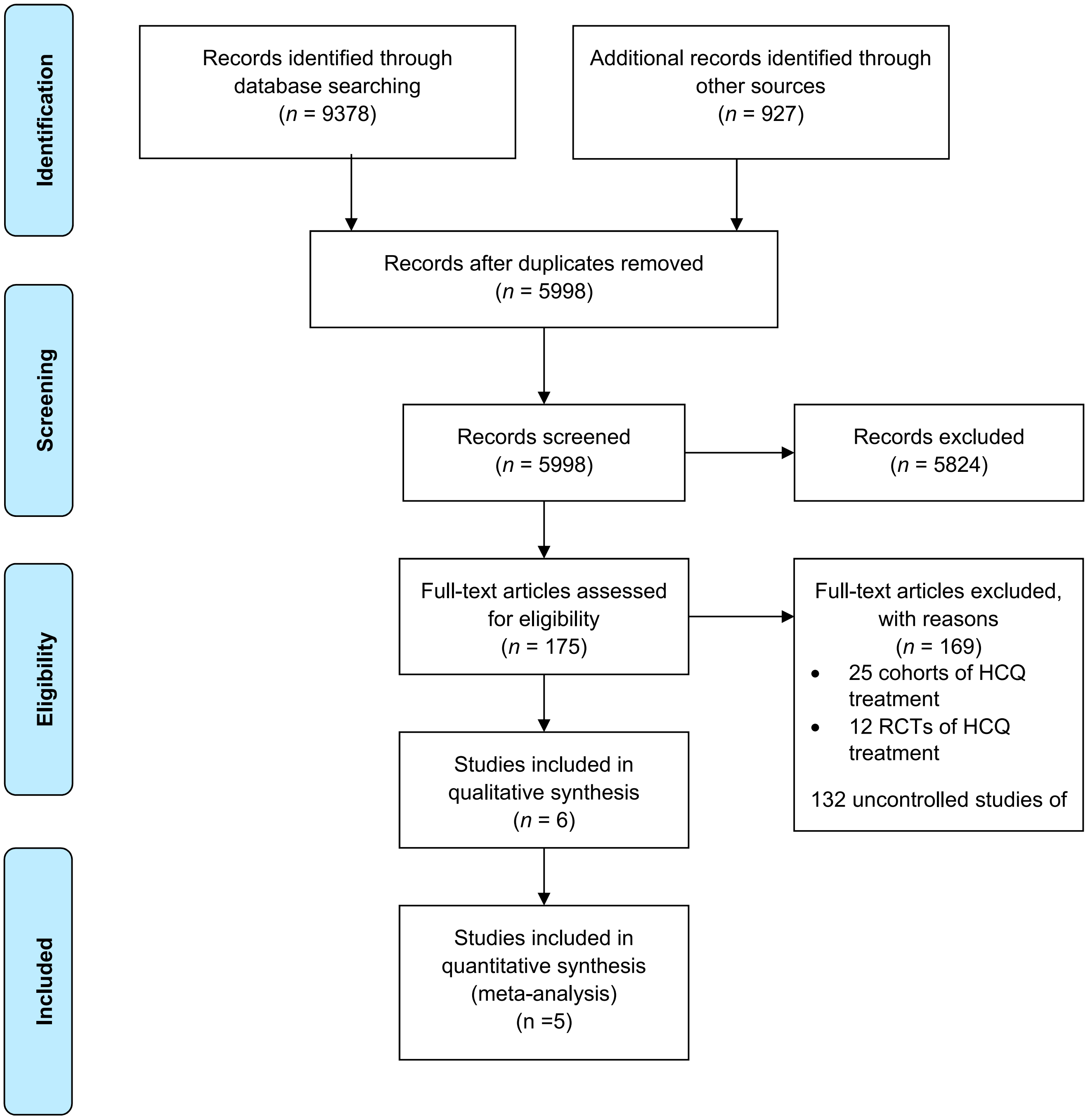

**Figure S2.** Risk of bias assessment of five randomized controlled trials.

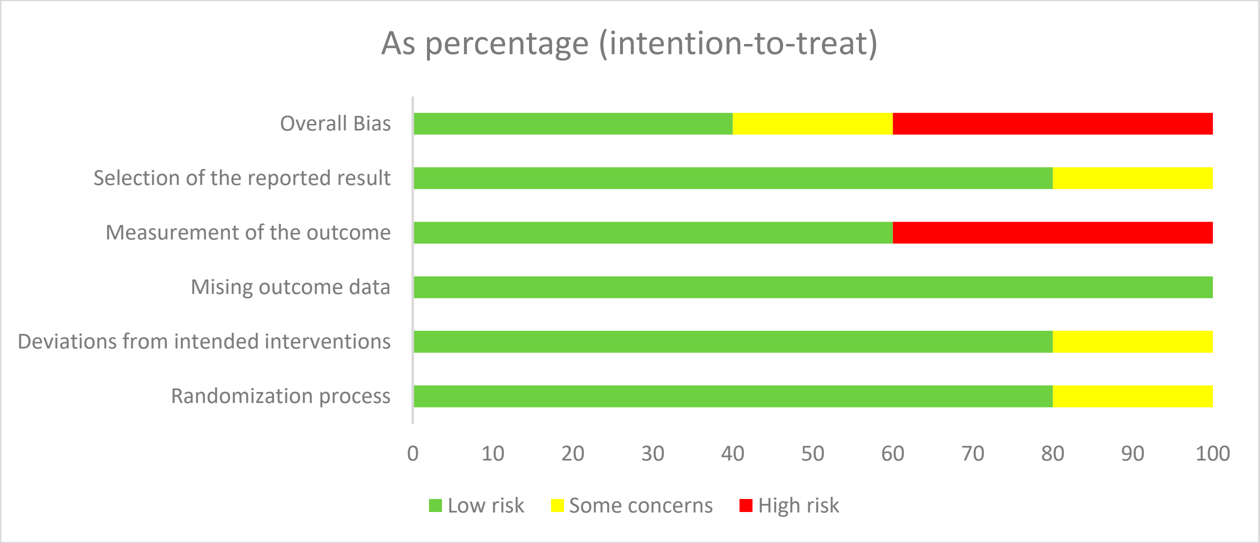


**Figure S3.** Detailed risk of bias assessment per randomized controlled trial.

| | Study ID | Experimental | Comparator | Outcome | Weight | Randomization process | Deviations from intended interventions | Missing outcome data | Measurement of the outcome | Selection of the reported result | Overall |
|---|---|---|---|---|---|---|---|---|---|---|---|
| 1 | Boulware | HCQ | Placebo folate | Incidence of confirmed or probable Covid-19 at 14 days | 1 | + | + | + | Ñ | + | Ñ |
| 2 | Rajasingham | HCQ twice weekly ( | Placebo folic acid | Confirmed or probable Covid-19 at 12w | 1 | + | + | + | Ñ | ? | Ñ |
| 3 | Abella | HCQ | Placebo | Incidence of lab confirmed SARS-CoV-2 infection at 8w | 1 | ? | ? | + | + | + | ! |
| 4 | Mitja | HCQ | Usual care | PCR-confirmed symptomatic Covid-19 within 14 days | 1 | + | + | + | + | + | + |
| 5 | Barnabas | HCQ | Placebo ascorbic acid | SARS-CoV-2 infection at 14 days | 1 | + | + | + | + | + | + |

+ Low risk
? Some concerns
Ñ High risk

**Figure S4.** Effect of prophylaxis with hydroxychloroquine on clinical worsening.

| Source | Hydroxychloroquine Events | Hydroxychloroquine Total | Control Events | Control Total | RR [95%-CI] | Weight |
|---|---|---|---|---|---|---|
| Boulware 2020 | 1 | 414 | 1 | 407 | 0.98 [0.06; 15.66] | 40.9% |
| Rajasingham 2020 | 0 | 989 | 0 | 494 | 1.00 [0.02; 63.88] | 18.2% |
| Barnabas 2020 | 1 | 407 | 1 | 422 | 1.04 [0.07; 16.52] | 40.9% |
| **Random effects model** | **2** | **1810** | **2** | **1323** | **1.01 [0.17; 5.92]** | **100.0%** |

Heterogeneity: $I^2 = 0\%$, $\tau^2 = 0$, $p = 1.00$

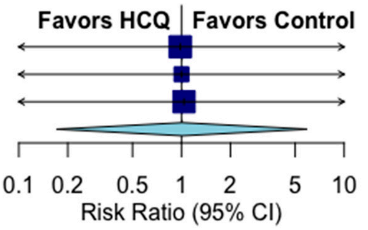


**Figure S5.** Effect of prophylaxis with hydroxychloroquine on severe adverse events.

| Source | Hydroxychloroquine Events | Hydroxychloroquine Total | Control Events | Control Total | RR [95%-CI] | Weight |
|---|---|---|---|---|---|---|
| Abella 2020 | 0 | 65 | 0 | 65 | 1.00 [0.02; 49.65] | 2.8% |
| Mitja 2020 | 14 | 1197 | 17 | 1300 | 0.89 [0.44; 1.81] | 86.1% |
| Barnabas 2020 | 2 | 407 | 2 | 422 | 1.04 [0.15; 7.33] | 11.1% |
| **Random effects model** | **16** | **1669** | **19** | **1787** | **0.91 [0.48; 1.75]** | **100.0%** |

Heterogeneity: $I^2 = 0\%$, $\tau^2 = 0$, $p = 0.99$

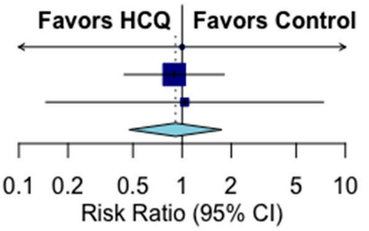


**Figure S6.** Effect of prophylaxis with hydroxychloroquine on adverse events.

| Source | Hydroxychloroquine Events | Hydroxychloroquine Total | Control Events | Control Total | RR [95%-CI] | Weight |
|---|---|---|---|---|---|---|
| **postexposure** | | | | | | |
| Mitja 2020 | 671 | 1197 | 77 | 1300 | 9.46 [7.58; 11.82] | 25.6% |
| Boulware 2020 | 140 | 349 | 59 | 351 | 2.39 [1.83; 3.11] | 25.4% |
| Barnabas 2020 | 66 | 407 | 46 | 422 | 1.49 [1.05; 2.11] | 25.0% |
| **Random effects model** | **877** | **1953** | **182** | **2073** | **3.25 [0.30; 35.44]** | **76.0%** |
| Heterogeneity: $I^2 = 98\%$, $\tau^2 = 0.9048$, $p < 0.01$ | | | | | | |
| **preexposure** | | | | | | |
| Abella 2020 | 29 | 65 | 17 | 65 | 1.71 [1.04; 2.78] | 24.0% |
| **Random effects model** | **29** | **65** | **17** | **65** | **1.71 [1.04; 2.78]** | **24.0%** |
| Heterogeneity: not applicable | | | | | | |
| **Random effects model** | **906** | **2018** | **199** | **2138** | **2.79 [0.72; 10.82]** | **100.0%** |
| Heterogeneity: $I^2 = 97\%$, $\tau^2 = 0.6963$, $p < 0.01$ | | | | | | |

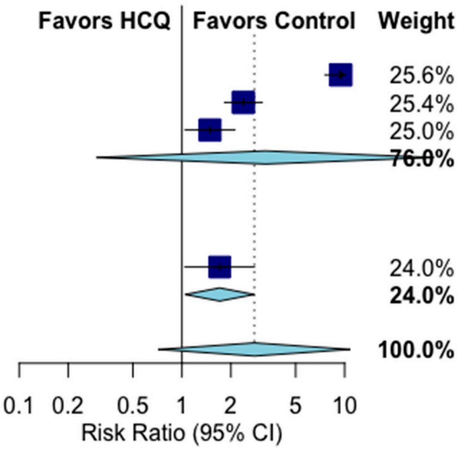

**Figure S7.** Effect of prophylaxis with hydroxychloroquine on diarrhea, abdominal pain or vomiting.

| Source | Hydroxychloroquine Events | Hydroxychloroquine Total | Control Events | Control Total | RR [95%-CI] | Weight |
|---|---|---|---|---|---|---|
| **postexposure** | | | | | | |
| Mitja 2020 | 510 | 1197 | 33 | 1300 | 16.78 [11.91; 23.66] | 21.7% |
| Boulware 2020 | 81 | 349 | 15 | 351 | 5.43 [ 3.19; 9.23] | 20.4% |
| Barnabas 2020 | 25 | 407 | 7 | 422 | 3.70 [ 1.62; 8.47] | 17.8% |
| **Random effects model** | **616** | **1953** | **55** | **2073** | **7.35 [ 1.03; 52.60]** | **59.8%** |
| Heterogeneity: $I^2 = 90\%$, $\tau^2 = 0.5396$, $p < 0.01$ | | | | | | |
| **preexposure** | | | | | | |
| Abella 2020 | 21 | 65 | 8 | 65 | 2.62 [ 1.25; 5.49] | 18.6% |
| Rajasingham 2020 | 140 | 989 | 35 | 494 | 2.00 [ 1.40; 2.85] | 21.6% |
| **Random effects model** | **161** | **1054** | **43** | **559** | **2.10 [ 0.54; 8.13]** | **40.2%** |
| Heterogeneity: $I^2 = 0\%$, $\tau^2 = 0$, $p = 0.51$ | | | | | | |
| **Random effects model** | **777** | **3007** | **98** | **2632** | **4.56 [ 1.58; 13.19]** | **100.0%** |
| Heterogeneity: $I^2 = 95\%$, $\tau^2 = 0.6452$, $p < 0.01$ | | | | | | |

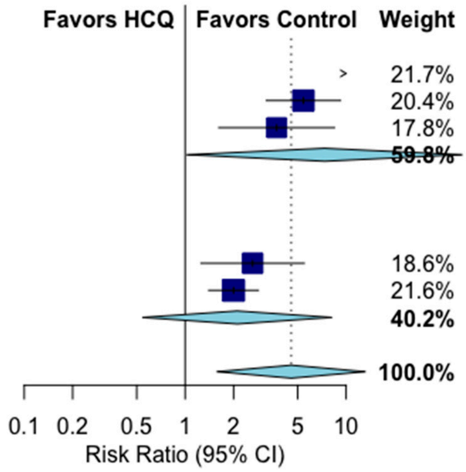


**Figure S8.** Effect of prophylaxis with hydroxychloroquine on headache.

| Source | Hydroxychloroquine Events | Hydroxychloroquine Total | Control Events | Control Total | RR [95%-CI] | Weight |
|---|---|---|---|---|---|---|
| **postexposure** | | | | | | |
| Mitja 2020 | 260 | 1197 | 32 | 1300 | 8.82 [6.16; 12.63] | 24.1% |
| Boulware 2020 | 13 | 349 | 8 | 351 | 1.63 [0.69; 3.89] | 22.0% |
| Barnabas 2020 | 5 | 407 | 7 | 422 | 0.74 [0.24; 2.31] | 20.4% |
| **Random effects model** | **278** | **1953** | **47** | **2073** | **2.38 [0.56; 10.12]** | **66.6%** |
| Heterogeneity: $I^2 = 92\%$, $\tau^2 = 1.4544$, $p < 0.01$ | | | | | | |
| **preexposure** | | | | | | |
| Abella 2020 | 0 | 65 | 4 | 65 | 0.11 [0.01; 2.02] | 10.6% |
| Rajasingham 2020 | 22 | 989 | 12 | 494 | 0.92 [0.46; 1.83] | 22.9% |
| **Random effects model** | **22** | **1054** | **16** | **559** | **0.52 [0.08; 3.25]** | **33.4%** |
| Heterogeneity: $I^2 = 48\%$, $\tau^2 = 1.0656$, $p = 0.17$ | | | | | | |
| **Random effects model** | **300** | **3007** | **63** | **2632** | **1.38 [0.39; 4.80]** | **100.0%** |
| Heterogeneity: $I^2 = 93\%$, $\tau^2 = 1.6511$, $p < 0.01$ | | | | | | |

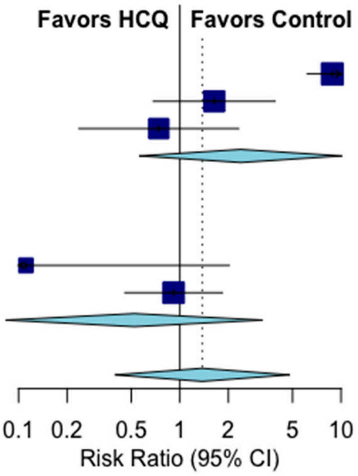

**PRISMA Checklist**

| Section/topic | # | Checklist item | Reported on page # |
|---|---|---|---|
| **TITLE** | | | |
| Title | 1 | Identify the report as a systematic review, meta-analysis, or both. | 1 |
| **ABSTRACT** | | | |
| Structured summary | 2 | Provide a structured summary including, as applicable: background; objectives; data sources; study eligibility criteria, participants, and interventions; study appraisal and synthesis methods; results; limitations; conclusions and implications of key findings; systematic review registration number. | 1 |
| **INTRODUCTION** | | | |
| Rationale | 3 | Describe the rationale for the review in the context of what is already known. | 1,2 |
| Objectives | 4 | Provide an explicit statement of questions being addressed with reference to participants, interventions, comparisons, outcomes, and study design (PICOS). | 2 |
| **METHODS** | | | |
| Protocol and registration | 5 | Indicate if a review protocol exists, if and where it can be accessed (e.g., Web address), and, if available, provide registration information including registration number. | – |
| Eligibility criteria | 6 | Specify study characteristics (e.g., PICOS, length of follow-up) and report characteristics (e.g., years considered, language, publication status) used as criteria for eligibility, giving rationale. | 2 |
| Information sources | 7 | Describe all information sources (e.g., databases with dates of coverage, contact with study authors to identify additional studies) in the search and date last searched. | 2 |
| Search | 8 | Present full electronic search strategy for at least one database, including any limits used, such that it could be repeated. | 2, Supplemental file |
| Study selection | 9 | State the process for selecting studies (i.e., screening, eligibility, included in systematic review, and, if applicable, included in the meta-analysis). | 2 |
| Data collection process | 10 | Describe method of data extraction from reports (e.g., piloted forms, independently, in duplicate) and any processes for obtaining and confirming data from investigators. | 3 |
| Data items | 11 | List and define all variables for which data were sought (e.g., PICOS, funding sources) and any assumptions and simplifications made. | 2 |

| | | | |
|---|---|---|---|
| Risk of bias in individual studies | 12 | Describe methods used for assessing risk of bias of individual studies (including specification of whether this was done at the study or outcome level), and how this information is to be used in any data synthesis. | 3 |
| Summary measures | 13 | State the principal summary measures (e.g., risk ratio, difference in means). | 3 |
| Synthesis of results | 14 | Describe the methods of handling data and combining results of studies, if done, including measures of consistency (e.g., $I^2$) for each meta-analysis. | 3 |

| Section/topic | # | Checklist item | Reported on page # |
|---|---|---|---|
| Risk of bias across studies | 15 | Specify any assessment of risk of bias that may affect the cumulative evidence (e.g., publication bias, selective reporting within studies). | 3 |
| Additional analyses | 16 | Describe methods of additional analyses (e.g., sensitivity or subgroup analyses, meta-regression), if done, indicating which were pre-specified. | 3 |
| **RESULTS** | | | |
| Study selection | 17 | Give numbers of studies screened, assessed for eligibility, and included in the review, with reasons for exclusions at each stage, ideally with a flow diagram. | 3; Figure S1 |
| Study characteristics | 18 | For each study, present characteristics for which data were extracted (e.g., study size, PICOS, follow-up period) and provide the citations. | 3,4; Table 1 |
| Risk of bias within studies | 19 | Present data on risk of bias of each study and, if available, any outcome level assessment (see item 12). | 6; Figures S3, S4 |
| Results of individual studies | 20 | For all outcomes considered (benefits or harms), present, for each study: (a) simple summary data for each intervention group (b) effect estimates and confidence intervals, ideally with a forest plot. | 6-8 |
| Synthesis of results | 21 | Present results of each meta-analysis done, including confidence intervals and measures of consistency. | 6-8; Figures |
| Risk of bias across studies | 22 | Present results of any assessment of risk of bias across studies (see Item 15). | 6, 8, Table 2 |
| Additional analysis | 23 | Give results of additional analyses, if done (e.g., sensitivity or subgroup analyses, meta-regression [see Item 16]). | 8 |
| **DISCUSSION** | | | |
| Summary of evidence | 24 | Summarize the main findings including the strength of evidence for each main outcome; consider their relevance to key groups (e.g., healthcare providers, users, and policy makers). | 11-13 |

| | | | |
|---|---|---|---|
| Limitations | 25 | Discuss limitations at study and outcome level (e.g., risk of bias), and at review-level (e.g., incomplete retrieval of identified research, reporting bias). | 12,13 |
| Conclusions | 26 | Provide a general interpretation of the results in the context of other evidence, and implications for future research. | 13 |
| **FUNDING** | | | |
| Funding | 27 | Describe sources of funding for the systematic review and other support (e.g., supply of data); role of funders for the systematic review. | 13 |
